# Cardiac Tamponade, an Unusual First Presentation of Systemic Lupus Erythematosus: A Case Report in a Rural Tertiary Hospital

**DOI:** 10.7759/cureus.27989

**Published:** 2022-08-14

**Authors:** Airenakho Emorinken, Mercy O Dic-Ijiewere, Hannah O Izirein

**Affiliations:** 1 Department of Internal Medicine, Irrua Specialist Teaching Hospital, Irrua, NGA

**Keywords:** echocardiography, pericardiocentesis, cardiac tamponade, pericardial effusion, systemic lupus erythematosus

## Abstract

Systemic lupus erythematosus (SLE) is a condition that manifests in a variety of ways. Although pericarditis and pericardial effusion are frequent cardiac manifestations of SLE, cardiac tamponade is rarely reported, especially as the initial manifestation of the disease. We describe a 38-year-old Nigerian lady who presented with three months of progressive dyspnea. She had intermittent fever, tachycardia, tachypnea, hypotension, jugular vein distension, and muffled heart sounds. Echocardiography confirmed cardiac tamponade. The ANA, anti-dsDNA, and anti-Sm antibodies were positive. She had a high ESR and low levels of blood complements. The diagnosis of SLE was established based on the 2019 EULAR/ACR classification criteria. She was treated with intravenous methylprednisolone, oral prednisolone, and hydroxychloroquine after undergoing an emergency echo-guided pericardiocentesis. She responded well to treatment, and she is currently being followed up on an outpatient basis. Clinicians should consider SLE as a differential when evaluating patients with pericardial effusion, as an accurate and timely diagnosis could be lifesaving.

## Introduction

Systemic lupus erythematosus (SLE) is a chronic multisystemic autoimmune disease with a variety of clinical manifestations. It is characterized by the formation of immune complexes and autoantibodies and can affect any organ system [1]. The disease is more prevalent among younger women, with a female-to-male prevalence ratio of approximately 10:1 [2]. Cardiac involvement is common and can be found in more than half of SLE patients [3,4]. Although pericarditis and pericardial effusion are the most common cardiac manifestations, cardiac tamponade, especially as the initial manifestation of the disease, is extremely uncommon [5,6]. In this case report, we describe a 38-year-old Nigerian woman whose initial manifestation of SLE was cardiac tamponade.

## Case presentation

A 38-year-old woman presented to the emergency unit with a three-month history of progressive breathlessness. In the past month, she had developed fatigue and intermittent fevers but had no joint pain. In the four days preceding her presentation, her dyspnea significantly worsened. There was associated chest pain. She had no history suggestive of heart failure, renal failure, or liver disease. She had no hair loss, oral ulcers, or rashes. She had no history of irrational speech or behavior. She was unaware of her hepatitis and retroviral status. She had no family history of autoimmune diseases. She was not a known patient with diabetes mellitus and/or hypertension. She doesn’t drink alcohol or smoke cigarettes.

On examination, she had a temperature of 37.8°C, a heart rate of 115 beats per minute, blood pressure of 90/60 mmHg, a respiratory rate of 28 breaths per minute, and oxygen saturation of 98% in room air. On cardiovascular examination, the heart sounds were muffled, and the jugular veins were distended. A respiratory examination revealed decreased breath sounds at the right lung base. An abdominal examination revealed hepatomegaly and ascites. Other examinations were unremarkable.

**Figure 1:**
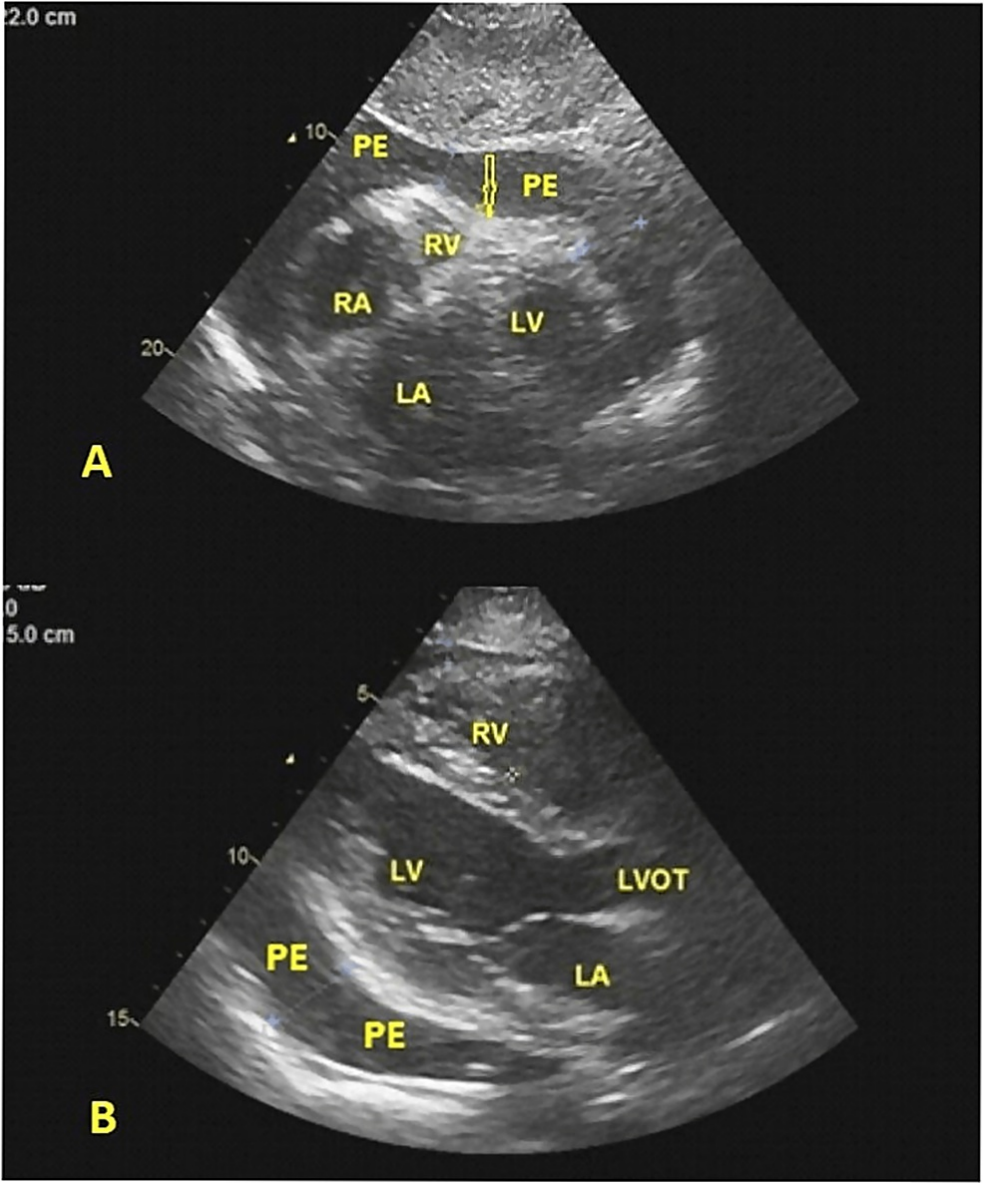
(A) Transthoracic 2-dimensional echocardiogram (subcostal view) showing a large pericardial effusion (PE) and diastolic collapse of the right ventricular free wall (arrow). (B) Transthoracic 2-dimensional echocardiogram (parasternal long axis view) showing pericardial effusion (PE) behind the left ventricular (LV) posterior wall.

The electrocardiogram showed sinus tachycardia and low voltages. The chest radiograph showed an increased cardiac silhouette. The transthoracic echocardiography revealed a large circumferential pericardial effusion with an echo-free space of 2.7 cm in the parasternal long axis view and 2.4 cm in the subcoastal view with the diastolic collapse of the right ventricle (Figure 1). There was also a significant inspiratory decrease in the mitral valve E wave velocity on pulse wave Doppler tracing, which confirmed a tamponade. 

Laboratory findings included: hemoglobin of 9.2 g/dL (12.0-18.5), leucocytes of 9.9 x 10^9^/L (4-11), platelets of 350 x 10^9^/L (150-450), erythrocyte sedimentation rate (ESR) of 72 mm/hour and a c-reactive protein (CRP) of 10 mg/L (0-7.4). Renal and liver function tests were normal. Urinalysis by dipstick was normal. Serological tests for HIV, hepatitis B, and C were negative. The Mantoux tuberculin skin test was negative. Blood and urine cultures were negative.

The patient underwent an emergency echocardiogram-guided pericardiocentesis, during which 800 mL of serosanguinous fluid was removed. Additionally, a pericardial drainage tube was kept in place to track drainage. This gave the patient much-needed symptomatic relief. Analyses of the pericardial fluid revealed 1400 leucocytes and 100 red blood cells per mm^3^. The cytology revealed inflammatory cells with no evidence of malignancy. Both bacteriologic cultures and GeneXpert were negative. Other pericardial fluid studies, such as protein, glucose, lactate dehydrogenase, and adenosine deaminase were unremarkable.

Additional laboratory testing was done to further determine the cause of the pericardial effusion. Anti-double stranded DNA (anti-dsDNA), anti-Smith (anti-Sm), and anti-SSA antibodies were all positive, as were antinuclear antibodies (ANA) at a titer of 1:1280 (<1:80). Direct Coombs testing was positive.

The patient had a score of 17 according to the 2019 European Alliance of Associations for Rheumatology/American College of Rheumatology (EULAR/ACR) classification criteria for SLE, confirming the diagnosis of SLE. The patient was started on intravenous methylprednisolone at a dose of 1 g/day and received a total of three doses, followed by oral prednisolone at a dose of 60 mg daily, which was subsequently tapered. She was also administered hydroxychloroquine orally. She had a favorable clinical response with a significant reduction in systemic congestion, and she is currently undergoing outpatient follow-up in the rheumatology and cardiology clinics.

## Discussion

The heart is frequently involved in SLE. Approximately half of SLE patients will experience cardiac complications [3,4]. Pericarditis, myocarditis, valve disorders, thrombosis, and conduction system abnormalities are among the most prevalent cardiac complications in SLE patients [2]. The most prevalent cardiac manifestation of SLE is pericarditis, which has been linked to poor survival. About 25% of all SLE patients develop symptomatic pericarditis at some point throughout the disease, most commonly in conjunction with concomitant pleuritis [7]. Nonetheless, autopsy investigations indicate a higher incidence of subclinical pericarditis [2,8]. Imaging examinations revealed that more than 30% of patients were found to have pericardial involvement [9]. Pericardial effusion is often small and hemodynamically insignificant in SLE [4]. Despite the prevalence of pericarditis, cardiac tamponade is thought to affect less than 1% of SLE patients, making it a rare and uncommon condition [10,11]. It is a strikingly unusual occurrence as an initial manifestation of SLE. Cardiac tamponade can occur in about 6% of patients with pericarditis [12].

Cardiac tamponade is a medical emergency that occurs when a pericardial effusion reaches a critical level, thereby limiting cardiac inflow and causing hemodynamic compromise. The clinical manifestations of tamponade are brought on by a decrease in cardiac output and systemic venous congestion. Cardiac tamponade as the first sign of SLE is very rare and only limited to case reports and series [6,10,13,14]. Typical symptoms and signs include dyspnea, orthopnea, chest pain, pulsus paradoxus, jugular venous distension, and hypotension [3,7]. The diagnosis is established by echocardiography. The core echocardiographic findings of pericardial tamponade are pericardial effusion, diastolic right ventricular collapse, systolic right atrial collapse, inferior vena cava plethora with minimal respiratory variation, and exaggerated respiratory cycle variation in mitral and tricuspid valve in-flow velocities [15]. The low incidence of tamponade despite the high prevalence of pericarditis in SLE may be attributable in part to the widespread use of steroids and nonsteroidal anti-inflammatory drugs (NSAIDs), which effectively reduce pericardial inflammation. 

In patients diagnosed with SLE, cardiac tamponade was more prevalent in women and those with anemia, renal disease, pleuritis, higher ESR values, and lower C4 levels [3]. In a series of 409 patients with SLE, of whom 24 developed tamponade, Goswami et al. found pleuritis, anti-nucleosome antibodies, and the size of pericardial effusion to be significant predictors of tamponade [16]. Our patient is a female with anemia, an elevated ESR, and pleuritis. Pericardial tamponade was confirmed with echocardiography, and the detection of ANA, anti-dsDNA, and anti-Sm antibodies in the serum confirmed the diagnosis of SLE. 

Nonsteroidal anti-inflammatory medications (NSAIDs) can be used to treat mild pericarditis. Cardiac tamponade in SLE should be treated with high-dose steroids and pericardiocentesis. Immunosuppressants such as hydroxychloroquine, mycophenolate mofetil, and azathioprine are also required [17,18]. Our patient had pericardiocentesis, high-dose steroids, and hydroxychloroquine. She had a positive therapeutic effect with this treatment without any further need for pericardiocentesis or pericardiectomy.

A pericardiocentesis is an excellent option for large effusions, but recurrence is common. A less invasive procedure called pericardial window can be used in this situation. Pericardiectomy is also an option if there is a future accumulation of pericardial fluid following pericardiocentesis and steroid therapy, and it is also beneficial for reducing future symptoms of constrictive pericarditis [17,18].

## Conclusions

Cardiac tamponade is a potentially fatal condition, and clinicians should consider SLE when evaluating patients with pericardial effusions. An accurate and timely diagnosis of this rare manifestation of SLE could be lifesaving. The rarity of cardiac tamponade as the first manifestation of SLE is highlighted in this case. Early detection of sero-immunological markers is critical for disease diagnosis.
